# A High-Speed Real-Time Sorting Method for Fabric Material and Color Based on Spectral-RGB Feature Fusion

**DOI:** 10.3390/s26051521

**Published:** 2026-02-28

**Authors:** Xin Ru, Yang Chen, Xiu Chen, Changjiang Wan, Jiapeng Chen

**Affiliations:** 1 College of Mechanical Engineering, Zhejiang Sci-Tech University, Hangzhou 310018, China; ruxin@zstu.edu.cn (X.R.); 2023220503012@mails.zstu.edu.cn (Y.C.);; 2 Zhejiang Sci-Tech University Longgang Research Institute Co., Ltd., Wenzhou 325000, China; 13758879635@163.com

**Keywords:** hyperspectral imaging, fabric material classification, waste textile sorting, automated recycling, intelligent manufacturing, vision-based classification, convolutional neural networks

## Abstract

A method for simultaneous classification of fabric material and color based on hyperspectral imaging and visual detection is proposed. Fabric material classification is performed using hyperspectral imaging (HSI) combined with a one-dimensional convolutional neural network (1D-CNN), while fabric color recognition is achieved using an red-green-blue (RGB) camera and a color classification model. Material and color features from the same fabric sample are matched to realize synchronous classification. Experiments were conducted on three fabric materials (cotton, polyester, and cotton–polyester blend) and eight colors. At a conveyor speed of 1 m/s, the sorting success rates reach 95.0% for cotton, 97.5% for polyester, and 85.0% for cotton–polyester blended fabrics. The proposed method demonstrates reliable performance for single-material fabrics and good industrial applicability for automated fabric sorting.

## 1. Introduction

### 1.1. Textile Market

Global textile production is dominated by polyester and cotton fibers, yet most textiles are landfilled or incinerated at the end of their life cycle. The demand for recycling is expected to increase continuously in the future. Since the 21st century, the global textile fiber output has grown at an average annual rate of 3.4%, reaching a total of 122 million tons in 2023 [1]. Cotton and polyester are the highest-yielding natural and chemical fibers, with outputs of 24.12 million tons and 71.7 million tons, respectively [1,2,3]. Over 97% of the raw materials required for global garment production are derived from virgin sources, with plastic-based fibers accounting for 63% and cotton for 26% [4].

Globally, the textile industry faces the dual challenges of resource waste and environmental pressure. Studies show that clothing utilization—defined as the average number of times a garment is worn before being discarded—has declined by 36% over the past 15 years. In the United States, the average usage is only a quarter of the global average, while in China, it has dropped by approximately 70% during the same period. This low utilization rate results in significant economic losses; annually, global consumers lose about $460 billion due to the premature disposal of still-usable clothing [4].

Some garments are discarded after only 7 to 10 wears [5]. Although some countries (e.g., Germany) achieve high recycling rates, the collected textiles are often exported to developing countries lacking recycling capabilities [6]. Consequently, most clothing ultimately ends up in landfills or is downcycled for low-value applications [7]. Globally, less than 1% of clothing materials are genuinely recycled back into garment production [8].

**Figure 1:**
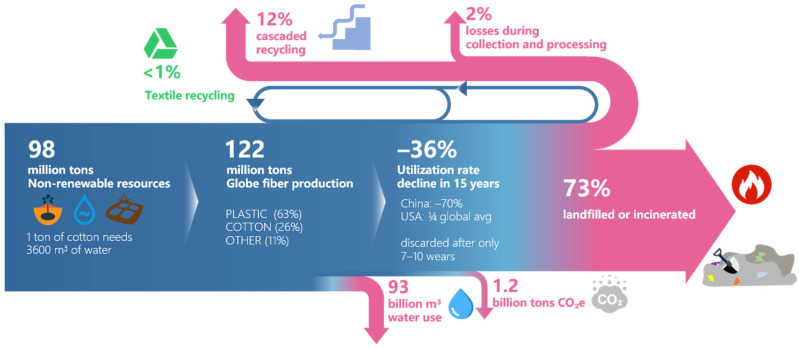
Textile cycle analysis.

The prevailing “linear” textile system not only incurs substantial economic losses but also imposes a severe burden on resources and the environment. The textile industry consumes approximately 98 million tons of non-renewable resources annually, including petroleum for synthetic fibers, fertilizers for cotton cultivation, and large quantities of chemicals used in dyeing and finishing [4]. Research indicates that up to 66% of waste textiles remain reusable. Extending the lifecycle of textiles plays a crucial role in resource conservation. Taking cotton as an example, producing 1 ton of raw cotton requires an average of 3600 cubic meters of water, and processing it into cotton fiber demands an additional 8500 cubic meters, accompanied by extensive use of pesticides and chemicals, leading to severe water pollution [6]. Furthermore, the overall textile production process consumes about 93 billion cubic meters of water annually, exacerbating water scarcity in some regions [4]. In 2015, the industry’s greenhouse gas emissions reached 1.2 billion tons of CO_2_ equivalent, surpassing the total emissions from all international flights and maritime shipping [9]. Additionally, the extensive use of hazardous chemicals in textile production poses significant risks to farmers, factory workers, and surrounding ecosystems (Figure 1).

### 1.2. Textile Detection Methods

Conventional methods for textile material identification, such as manual tactile and visual inspection, burning tests, microscopy, and dissolution, often rely on empirical judgment, are somewhat destructive, and exhibit low efficiency, making them unsuitable for large-scale, rapid detection [10]. Thermal analysis and laser Raman spectroscopy can provide certain molecular-level information, but in practical textile sorting scenarios, they are often constrained by detection speed, sample handling requirements, and equipment costs [11,12]. In contrast, near-infrared (NIR) spectroscopy offers advantages such as speed, non-destructiveness, and the potential for online detection, making it the primary choice for this study [13].

### 1.3. NIR-HSI Research Status

Huang et al. (2022) used hyperspectral imaging with a 1D-CNN to classify textile fibers, achieving 98.6% accuracy, outperforming many traditional methods [14]. Eghtedari et al. (2025) applied a bandpass-filtered NIR camera and an intensity metric to distinguish cotton, polyester, and carbon-black dyed areas, demonstrating NIR imaging for non-destructive material identification [15]. Industrial applications have also been explored: Becker et al. (2024) validated polyester textile waste sorting with NIR spectroscopy under industrial conditions [16], while Faghih et al. (2025) highlighted AI-based sorting’s strengths in pure materials but limitations for blended fabrics, suggesting combined imaging and spectral features [17]. Kainz et al. (2025) proposed supervised and unsupervised deep learning on NIR hyperspectral data for automatic textile classification [18], and Sormunen et al. (2025) extended this to quantify blended fabric compositions [19].

Although hyperspectral imaging provides high material specificity, traditional RGB-based visual inspection remains widely used for real-time defect detection. Li et al. (2013) demonstrated online fabric defect inspection with smart visual sensors [20], and recent YOLO-based methods achieved real-time industrial detection [21]. High-level reviews have also highlighted hyperspectral imaging’s broader applicability for non-destructive material identification [22].

Despite these advances, Wang et al. (2025) noted that deep learning for hyperspectral classification still shows variability across datasets, indicating limited robustness and generalization [23]. Industrial implementations are few but promising, such as the Specim–PICVISA pilot for hyperspectral-based automatic textile sorting [24].

### 1.4. Research Objectives and Problems

Hyperspectral near-infrared technology has previously been applied to textile identification and analysis. However, most existing studies rely on NIR spectrometers, primarily targeting fiber composition or foreign matter identification, and employ traditional machine learning models. These methods are not only limited to material discrimination, lacking the ability to distinguish color, but are also difficult to apply in high-speed sorting environments [14,15,16,18,19]. Therefore, this study proposes a detection framework that combines RGB and hyperspectral cameras, utilizing deep learning methods to achieve joint recognition of textile material and color, and explores its application in large-scale industrial sorting scenarios. We believe this method can provide efficient and reliable technical support for textile recycling and reuse.

## 2. Materials and Methods

In industrial textile recycling, waste fabrics pass through multiple processing stages before entering automated sorting systems. The fabric samples used in this study originate from typical post-consumer and post-industrial textile waste streams and are representative of real industrial recycling conditions. To ensure practical relevance, three commonly encountered fabric categories—cotton, polyester, and cotton–polyester blended fabrics—were selected for system development and experimental validation.

Prior to automated detection, waste textiles undergo a series of preprocessing operations, including cleaning to remove surface contaminants, removal of non-textile components (e.g., buttons and labels), and size reduction through cutting or shredding. These steps aim to eliminate foreign materials, standardize the physical state of the fabrics, and improve the reliability of subsequent optical inspection.

After preprocessing, the fabric pieces are segmented and mechanically fluffed, resulting in loosely distributed pieces with irregular shapes and varying sizes. At this stage, the fabrics can be arranged in a single layer on a conveyor belt, avoiding stacking and folding, which is essential for high-speed online detection. The waste fabric detection system proposed in this study is designed to operate specifically at this post-preprocessing stage, where fabrics have completed physical preparation but have not yet been assigned to material-specific recycling or reuse pathways.

**Figure 2:**
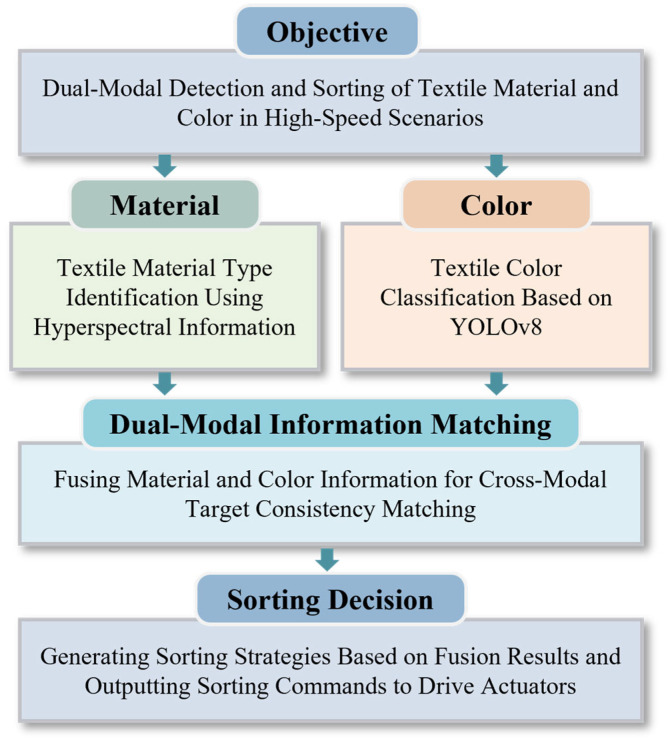
Overall workflow.

Under these operating conditions, the system performs real-time identification of fabric material and color and provides accurate spatial information for downstream robotic grasping and sorting. Based on this industrial background, the overall architecture of the waste fabric detection system is introduced in the following subsections (Figure 2).

### 2.1. Waste Fabric Sorting System

#### 2.1.1. Sorting Production Line

**Figure 3:**
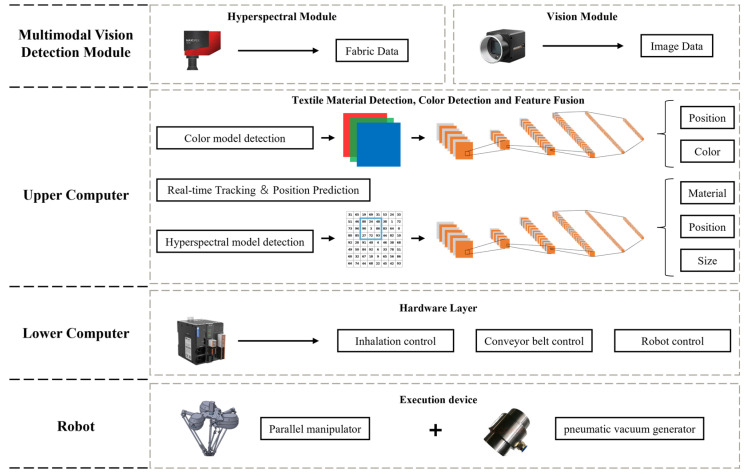
Schematic diagram of the sorting system.

As shown in Figure 3, the sorting system consists of five main components: the Hyperspectral Imaging Module, the Vision Module, the Parallel Robot Manipulator, the Host Controller (Upper Computer), and the Slave Controller (Lower Computer). The Hyperspectral Imaging Module comprises one HX-179 hyperspectral camera (spectral range: 900–1700 nm, number of spectral bands: no less than 224) (Hangzhou Gaopu Imaging Technology Co., Ltd., Hangzhou, China), two rows of halogen lamps (6 lamps total, 400 W each), a light-tight enclosure, and an exhaust device. It is primarily used for collecting fabric spectral information and achieving material identification. The Vision Module consists of one Hikvision MV-CS016-10UC RGB camera (resolution: 1.6 megapixels, maximum frame rate: 249.1 fps) (Hangzhou Hikvision Digital Technology Co., Ltd., Hangzhou, China), a light-tight enclosure, and an LED rectangular light source, used for image acquisition and target detection. The Actuator Section is composed of a parallel manipulator and a vacuum suction system based on pneumatic negative pressure. The vacuum suction system includes a suction base, a pneumatic negative pressure generator, and a vacuum suction device, responsible for picking and sorting waste fabrics.

The Host Controller employs an industrial computer equipped with an AMD Ryzen 7500F processor, an NVIDIA RTX A5000 GPU, and 48 GB of RAM, running the Windows 10 operating system. It is responsible for receiving data from the hyperspectral and vision modules, executing target detection and positioning, spectral information analysis, actuator control, and motion planning. The Slave Controller (PLC) is connected to the manipulator driver and production line equipment, realizing real-time control of robot movements by issuing control signals. During experiments, considering the high fault tolerance of the vacuum suction device, the system adopts a sorting method based on planar grasping. This method keeps the height of the suction end constant during operation and does not rely on image depth information, thereby simplifying the perception and planning processes, making it more suitable for high-speed industrial sorting application scenarios. For this purpose, a high-frame-rate RGB camera is selected for the vision module and installed inside the light-tight enclosure above the conveyor belt, with the camera lens parallel to the conveyor belt, forming a typical “eye-to-hand” configuration.

As shown in Figure 3, the control and communication framework of the sorting system consists of the hyperspectral detection module, the vision detection module, the host computer control system, and the slave computer (PLC) control system. The hyperspectral camera is connected to the first host computer via a CameraLink frame grabber for high-speed acquisition and transmission of spectral data. Data exchange between the first and second host computers is realized through Socket communication based on the TCP/IP protocol, transmitting spectral detection results and material identification information. The Hikvision RGB camera is connected to the second host computer via a USB 3.0 interface, responsible for real-time image data acquisition and target detection. After fusing the hyperspectral and vision detection information, the second host computer establishes a communication connection with the slave controller (PLC) via Ethernet, employing the OPC UA protocol for data interaction and issuing sorting control commands to the PLC. Upon receiving the commands, the PLC controls the parallel manipulator driver, vacuum suction device, conveyor belt, and other execution equipment through I/O signals, thereby achieving real-time control of sorting actions and closed-loop operation of the system.

#### 2.1.2. Sorting Control and Analysis

**Figure 4:**
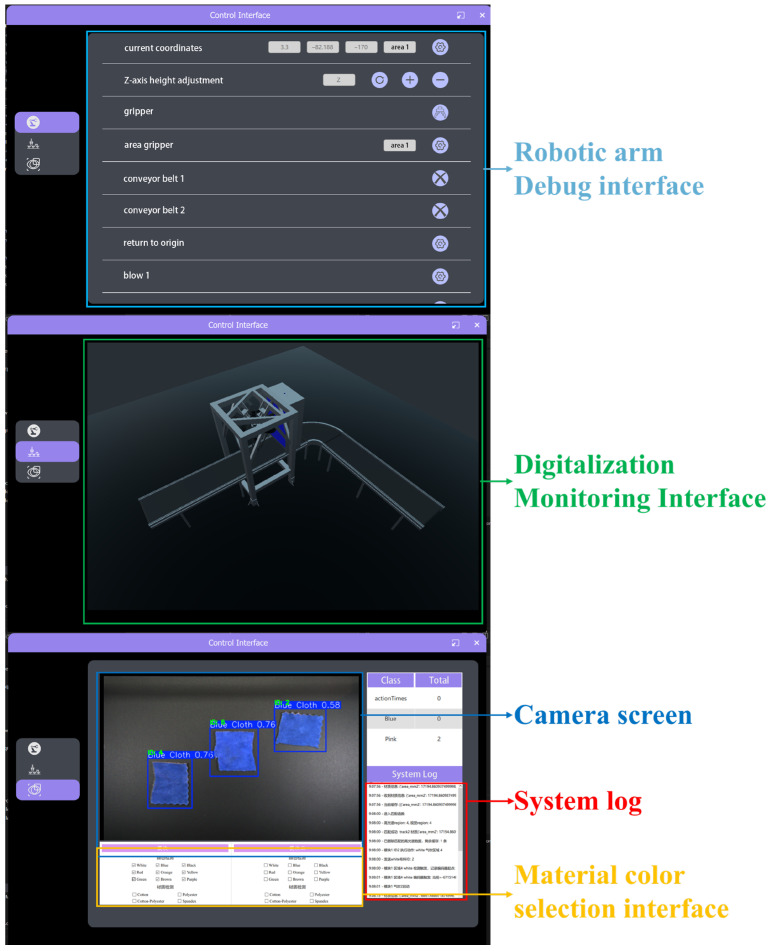
Software interface.

The entire system’s software framework is developed on the Python (3.12.9) platform and runs in a Windows 10 computer environment. The hyperspectral and vision modules are encapsulated as independent threads, using PyTorch (2.5.1) for neural network inference, OpenCV for image processing, and Socket for cross-host communication. The program adopts a modular and multi-threaded design, significantly enhancing the system’s real-time performance and stability, and providing a solid software foundation for subsequent algorithm expansion and parameter optimization (Figure 4).

The system adopts a collaborative architecture consisting of two host computers and a programmable logic controller (PLC). The first host computer, developed based on the manufacturer’s hyperspectral detection platform with customized algorithm enhancements, is responsible for hyperspectral data acquisition, spectral feature extraction, and fabric material identification. Additional modules for fabric contour extraction and dimension calculation are integrated to obtain spatial position and shape information. The second host computer, implemented in Python, receives hyperspectral and RGB vision results, performs material–color feature matching, target recognition, and sorting decision-making, and transmits sorting commands to the PLC via the OPC UA protocol. The PLC controls the manipulator and actuators to execute fabric grasping and sorting. The software adopts a modular design with a Qt/QML-based graphical interface, providing real-time result visualization, system parameter configuration, and sorting log management, thereby enhancing system operability and industrial applicability. As shown in Figure 4, the human–machine interface enables intuitive monitoring of the detection and sorting process.

### 2.2. Region of Interest (ROI) and Scanline Trigger Mechanism

**Figure 5:**
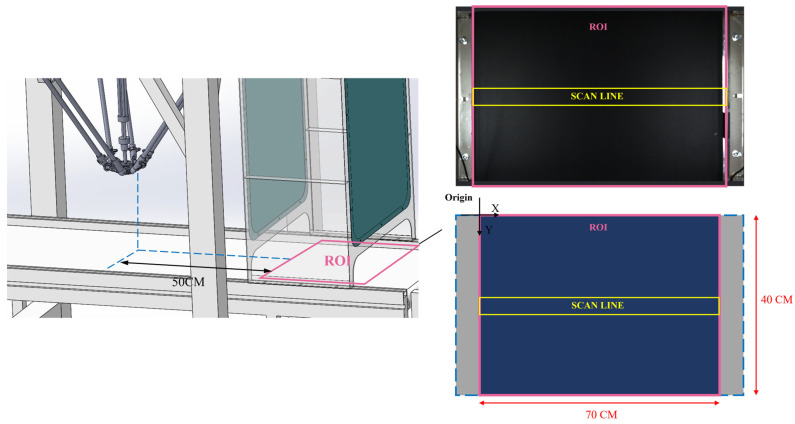
ROI diagram.

As shown in Figure 5, the system’s ROI covers the entire camera image. The image coordinate system is accurately mapped to the conveyor belt world coordinate system through a traditional checkerboard calibration method. The ROI serves as the primary coordinate system for target detection and multi-frame tracking. The downstream robot grasping area is offset approximately 0.5 m forward along the conveyor belt direction relative to this coordinate origin. As shown in the figure, the system’s ROI covers the entire camera image. Its image coordinate system is accurately mapped to the conveyor belt world coordinate system through a traditional checkerboard calibration method. The ROI is used as the main coordinate system for target detection and multi-frame tracking, while the downstream robot grasping area is offset approximately 0.5 m forward along the conveyor belt direction relative to this coordinate origin.

To ensure the accuracy of grasping timing in high-speed operation scenarios, the system designs a scanline pixel region within the ROI as a temporal reference point for action triggering. This scanline is located in the central area of the image, with a height of approximately 70 pixels and a width covering the entire image (640 pixels). It is important to emphasize that the scanline is not used for target classification, validity judgment, or tracking stability assessment; these steps are all performed within the entire ROI range through YOLO detection and a greedy IoU matching-based multi-frame tracking mechanism. The sole function of the scanline is to trigger the subsequent grasping process when a target’s center point first enters this pixel band (while maintaining downward motion). At this moment, the system records the current encoder position and, combined with the target’s region information and hyperspectral material identification result, sends a unique processing instruction to the actuator. This mechanism ensures each target is triggered only once and synchronizes the grasping action with the target’s actual spatial location in time.

The physical length corresponding to the scanline can be calculated based on camera calibration results as:

(1) $$W_{sl}=\frac{h_{sl}}{H_{img}}\cdot H_{FOV}$$

where *h_sl_* = 70 px is the scanline height, *H_img_* = 448 px is the total image height, and *H_FOV_* is the physical field-of-view width of the camera along the conveyor belt direction.

The dwell time of the target center within the scanline is:

(2) $$T_{sa}=\frac{W_{sl}}{V_{cb}}$$

The corresponding effective number of frames is:

(3) $$F_{sa}=FPS\times T_{sa}$$

The system’s camera frame rate is 55 FPS, and the conveyor belt speed is 1 m/s. Therefore, a target typically remains within the scanline region for only 1–2 frames. Classification and stable tracking are completed across multiple consecutive frames within the entire ROI, while the scanline solely performs “temporal triggering.”

To avoid repeated triggering, the system issues a grasping signal only when all the following conditions are simultaneously satisfied. According to the code logic, these conditions can be mathematically defined as a set:

(4) $$S=\left\{i\left|\begin{array}{c} hitsi\geq H_{min}\\ y_{sl}^{min}<y_{i}<y_{sl}^{max}\\ y_{i}(t)-y_{i}(t-1)>0\\ i\notin S_{sent} \end{array}\right.\right\}$$

where:

- *hits_i_*: The number of consecutive valid frames in which target *i* is detected within the ROI;
- *H_min_* = 3: The minimum number of valid detections (min_hits);
- $y_{sl}^{min}$, $y_{sl}^{max}$: The lower and upper boundaries of the scanline (180 px and 250 px);
- $y_{i}(t)-y_{i}(t-1)>0$: The target is moving downward;
- *S_sent_*: The set of targets that have already triggered a signal.

When target *i* satisfies all conditions in set *S*, the system records the current encoder value and triggers the grasping process. This process simultaneously incorporates the color recognition result and the hyperspectral material judgment result to ensure the final action is unique and correct.

Since the scanline serves only as a temporal trigger, its position and dimensions can be adjusted based on conveyor belt speed, camera position, calibration parameters, and inference speed to adapt to different industrial scenarios and maintain high robustness.

### 2.3. Hyperspectral Material Classification Model

#### 2.3.1. Hyperspectral Dataset Construction and Preprocessing

**Figure 6:**
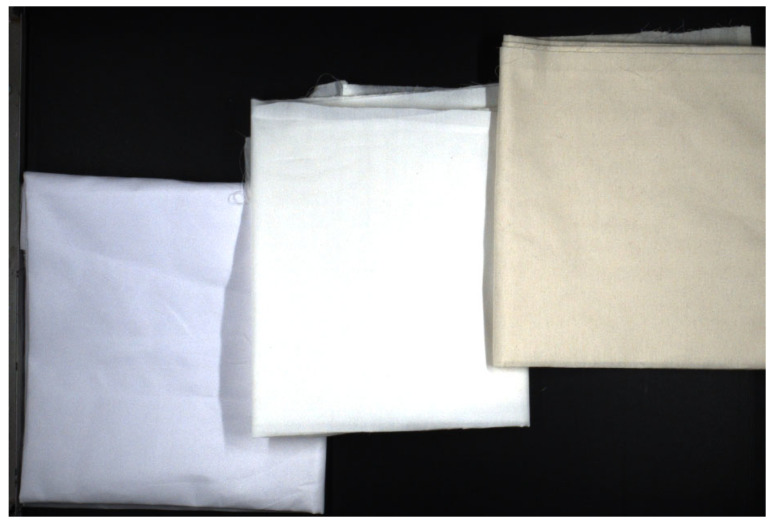
Fabric samples used in the experiment. From left to right: 100% polyester, cotton–polyester blend, and 100% cotton.

The material identification module in this study is based on near-infrared hyperspectral data acquired using a line-scan hyperspectral camera. To ensure the reliability and representativeness of the dataset, textile material samples were uniformly provided by the Zhejiang Academy of Quality Science, covering three typical material categories commonly encountered in industrial textile sorting scenarios: cotton, polyester, and cotton–polyester blends. All samples were calibrated and certified by professional institutions, guaranteeing the correctness of the ground-truth material labels (Figure 6).

The hyperspectral camera operates in a line-scan acquisition mode, capturing spectral information of an entire pixel line at each exposure. Multiple discrete wavelength bands were sampled within the near-infrared range of 900–1700 nm. Compared with conventional three-dimensional hyperspectral data cubes (H × W × Bands), the line-scan acquisition mechanism is more suitable for high-speed conveyor belt applications, as it enables real-time spectral data acquisition when textile targets pass through the sensing position.

Under uniform halogen lamp illumination, static spectral scans were performed only during the system calibration stage, including white reference, dark reference, and background acquisition, using the acquisition software provided by the camera manufacturer to ensure radiometric consistency and minimize spectral distortion caused by illumination inconsistency. During actual textile sample collection, hyperspectral data were acquired online while the textile samples were moving on the conveyor belt, corresponding to realistic operating conditions of the industrial sorting system.

For each textile sample, a representative region was manually selected from the dynamically acquired hyperspectral data cube. The spectral feature was then constructed by averaging pixel-level reflectance values within the selected region. This averaging operation was adopted as an engineering strategy to suppress random sensor noise and local spectral fluctuations induced by fabric texture variations, wrinkles, slight motion blur, and minor surface contamination during online acquisition. Rather than artificially curating idealized spectra, this aggregation provides a stable representation of the dominant material spectral characteristics while retaining robustness to practical disturbances encountered in high-speed conveyor environments.

Each hyperspectral sample can be represented as a one-dimensional spectral vector composed of discrete wavelength reflectance values:

(5) $$s=[s(\lambda_{1}),s(\lambda_{2}),...,s(\lambda_{N})]\in R^{N}$$

where $s(\lambda_{i})$ denotes the spectral reflectance value at the wavelength $\lambda_{i}$, and *N* represents the number of sampled spectral bands.

Prior to model training, all spectral vectors were normalized using z-score standardization to eliminate scale differences among wavelength bands and suppress noise effects. The final hyperspectral material dataset consisted of 904 valid spectral samples across the three material categories.

#### 2.3.2. One-Dimensional CNN Architecture for Spectral Feature Extraction

To effectively exploit the intrinsic correlation along the spectral dimension, a one-dimensional convolutional neural network (1D-CNN) was adopted for hyperspectral material classification. The normalized one-dimensional spectral vector defined in Equation (5) was used as the network input, with a single input channel corresponding to the reflectance sequence along the wavelength axis.

The proposed 1D-CNN architecture consists of four convolutional blocks. The first two convolutional layers employ convolution kernels with a size of 5 to capture local spectral patterns, while the subsequent two layers adopt kernel sizes of 3 to extract higher-level spectral features. Each convolutional layer is followed by batch normalization and a ReLU activation function to enhance training stability and nonlinear representation capability. Max-pooling operations are applied after the first three convolutional blocks to progressively reduce the spectral dimensionality and improve robustness to spectral variations.

In the final convolutional block, an adaptive average pooling layer is introduced to aggregate spectral features along the wavelength dimension, enabling the network to accommodate variations in spectral length. The extracted features are then flattened and processed by three fully connected layers to generate high-level feature representations and perform material classification. Dropout layers with different rates are incorporated throughout the network to alleviate overfitting. Finally, a softmax classifier is employed in the output layer to produce the probability distribution over the three material categories. The detailed network architecture is summarized in Table 1.

#### 2.3.3. Model Training Strategy and Implementation Details

The constructed hyperspectral dataset was randomly divided into training, validation, and testing subsets with a ratio of 70%, 15%, and 15%, respectively. Stratified sampling was adopted to ensure a balanced distribution of the three material categories across all subsets.

Model training was performed using the cross-entropy loss function and the Adam optimizer, with an initial learning rate of 0.001 and a batch size of 32. The maximum number of training epochs was set to 100. To improve convergence behavior and generalization performance, a learning rate scheduling strategy based on validation loss was employed during training. In addition, an early stopping mechanism was adopted to prevent overfitting by terminating training when the validation performance no longer improved.

The final model parameters were selected according to the highest validation accuracy. All experiments were implemented using the PyTorch deep learning framework on a GPU-enabled workstation.

### 2.4. Textile Color Classification Model

The textile color recognition dataset used in this study was constructed from video sequences captured by a high-speed area-scan RGB camera installed inside a closed, light-tight industrial enclosure. To ensure stable and repeatable color perception, the system employed CRI90 high–color-rendering LED strips as the illumination source. The LED light source was driven by a four-channel industrial controller with 8-bit PWM intensity control (0–255), allowing precise and repeatable adjustment of illumination intensity. During standard data acquisition, the camera exposure time and gain were kept constant, and uniform illumination was maintained across the imaging area.

**Figure 7:**
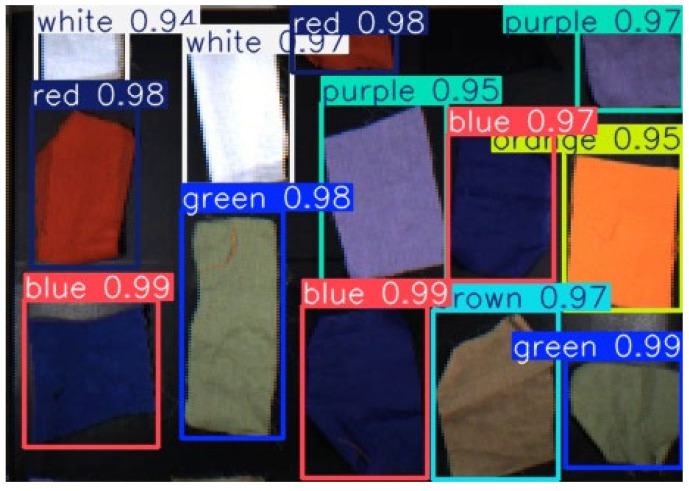
Color dataset samples.

All fabric samples were continuously imaged as they moved through the field of view on the conveyor belt. The acquired video streams were split into individual frames to form the color recognition dataset. Due to the inherently low reflectance of black fabrics in the visible spectrum and their strong visual similarity to the conveyor belt background under RGB imaging, black-colored samples were excluded from the color classification task. Consequently, the dataset covers eight common base colors encountered in industrial textile sorting applications: white, red, green, blue, yellow, brown, orange, and purple (Figure 7).

The extracted frame images were manually annotated by selecting valid fabric regions using the LabelImg tool to ensure accurate color labeling. Prior to model training, all images were uniformly resized to 640 × 640 pixels and underwent normalization and color channel standardization to match the input requirements of the YOLOv8n network.

To improve the robustness of the color classification model under variations in fabric texture, surface wrinkles, and moderate brightness changes, multiple data augmentation strategies were applied during training. These included brightness and contrast perturbation, random cropping, translation, and small-angle rotation. The color classification model was implemented based on the lightweight YOLOv8n architecture, which preserves real-time inference performance while maintaining stable low-frequency feature representations critical for color discrimination. Model training was performed using the AdamW optimizer with a cosine learning rate decay schedule, and the cross-entropy loss function was adopted for color category optimization.

The final color dataset comprises 222 valid image samples across eight color categories. The dataset was divided into training, validation, and test subsets following an approximate ratio of 7:1.5:1.5. Stratified sampling was employed to ensure balanced class representation across the three subsets. The detailed sample distribution and dataset splits for each color category are summarized in Table 2. Experimental results demonstrate that the trained model achieves stable classification performance on the validation set and is capable of real-time color prediction in high-speed conveyor belt scenarios. Combined with the hyperspectral material classification module, the color recognition model provides essential semantic information for subsequent sorting decisions.

### 2.5. Textile Localization

**Figure 8:**
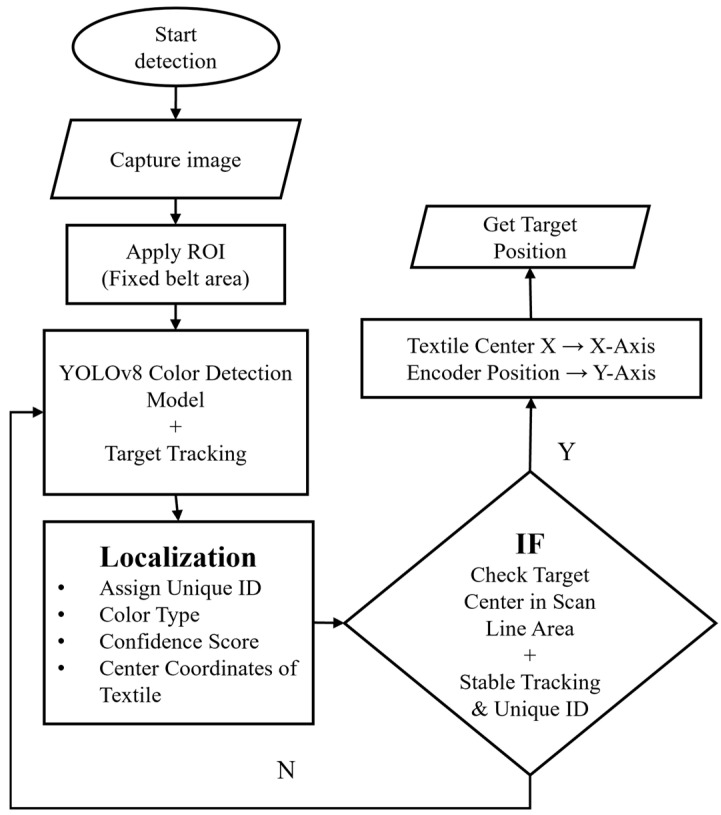
ML flowchart explaining object detection using the ML model.

The textile localization module of this system combines target detection, a lightweight target tracking algorithm based on maximum IoU, and an encoder position triggering mechanism to achieve robust estimation of textile positions in high-speed conveyor belt environments (Figure 8). Unlike traditional timestamp-based localization methods, this study employs the pulse sequence output by the conveyor belt encoder as a unified spatial reference, directly binding localization results to the conveyor belt travel coordinates. This approach avoids the accumulation of position errors caused by camera exposure delays and system processing delays.

First, the YOLOv8n model performs detection on RGB images, outputting bounding boxes for the textiles. The top-left and bottom-right pixel coordinates of a bounding box are denoted as (*x*_1_, *y*_1_) and (*x*_2_, *y*_2_), respectively. Its image center coordinates are calculated as follows:

(6) $$C_{x}^{img}=\frac{x_{1}+x_{2}}{2},C_{y}^{img}=\frac{y_{1}+y_{2}}{2}$$

To ensure that detection results across multiple frames stably correspond to the same object, this study employs a greedy association algorithm based on maximum IoU (Intersection-over-Union) for target tracking. The system calculates IoU between detection boxes in the current frame and the previous frame and maintains target ID continuity through a maximum matching principle. This method has low computational cost and strong real-time performance, making it suitable for high-speed online detection scenarios.

After obtaining a stable pixel-level center position, it is mapped to the conveyor belt plane coordinate system using a pre-calibrated 2D affine mapping matrix *A* to achieve engineering-level geometric localization:

(7) $$\left[\begin{array}{c} X_{w}\\ Y_{w} \end{array}\right]=A\left[\begin{array}{c} C_{x}^{img}\\ C_{y}^{img}\\ 1 \end{array}\right]$$

Here, A is constructed from the pixel coordinates and actual coordinates of three calibration points on the conveyor belt surface, stably describing the linear mapping from the image plane to the conveyor belt coordinate system.

To evaluate the mapping accuracy, additional validation points not used in the affine fitting process were introduced, and the resulting pixel-to-belt mapping error exhibited a mean absolute error of approximately 4–6 mm, with a maximum error below 10 mm.

Unlike conventional methods that rely on time-triggering for trajectory prediction, this study adopts an encoder position-triggering mechanism. The encoder outputs displacement pulses of the conveyor belt in real time. When an object’s center crosses the encoder count interval corresponding to a preset scanning position, the system records the object’s position in real time:

(8) $$Y_{enc}=f(N_{enc})$$

where *N_enc_* is the cumulative pulse count from the encoder, and *f*(·) is a linear conversion function derived based on the encoder’s pulses per revolution and the conveyor belt roller diameter. This position triggering method ensures the localization reference comes directly from the conveyor belt itself, unaffected by camera frame rate fluctuations or delays. As a result, the longitudinal localization of textile targets is primarily determined by the encoder measurements, while the affine mapping mainly provides lateral geometric alignment.

Finally, the system maintains a unique ID for each target and integrates the continuous trajectory from target tracking, the color category output by the RGB model, the material category output by the hyperspectral model, and the spatial coordinates obtained from encoder triggering. When an object’s center enters the sorting window area, the host computer sends the target ID, color category, material category, and final spatial position to the sorting execution terminal via the OPC UA protocol to drive pneumatic valves or mechanical actuators for precise sorting. It is worth noting that the downstream sorting module employs a vacuum-based end effector with a suction cup diameter of 52 mm, which provides inherently higher positional tolerance compared with gripper-type end effectors. As a result, moderate geometric localization errors introduced by affine mapping do not directly lead to grasping or sorting failure, and the achieved localization accuracy is sufficient for reliable system operation.

### 2.6. Hyperspectral Image Stitching Algorithm

This study employs a line-scan hyperspectral camera for row-by-row acquisition of textiles. The camera acquires complete single-row spectral data per exposure. As the conveyor belt moves, new scan rows are continuously received, allowing the construction of a 2D hyperspectral image of the target along the time dimension. The hyperspectral host computer receives and parses the region information for each row in real time. Each row segment is recorded in the structure:

(t,s,e,c)

where *t* is the timestamp, *s* and *e* are the starting and ending columns of the row segment, respectively, and *c* is the material category label for that row segment.

During the scanning process, the system dynamically associates the sequentially arriving row data by maintaining an active region buffer. Let the center position of a row segment be:

(9) $$c_{ent}=\frac{s+e}{2}$$

**Figure 9:**
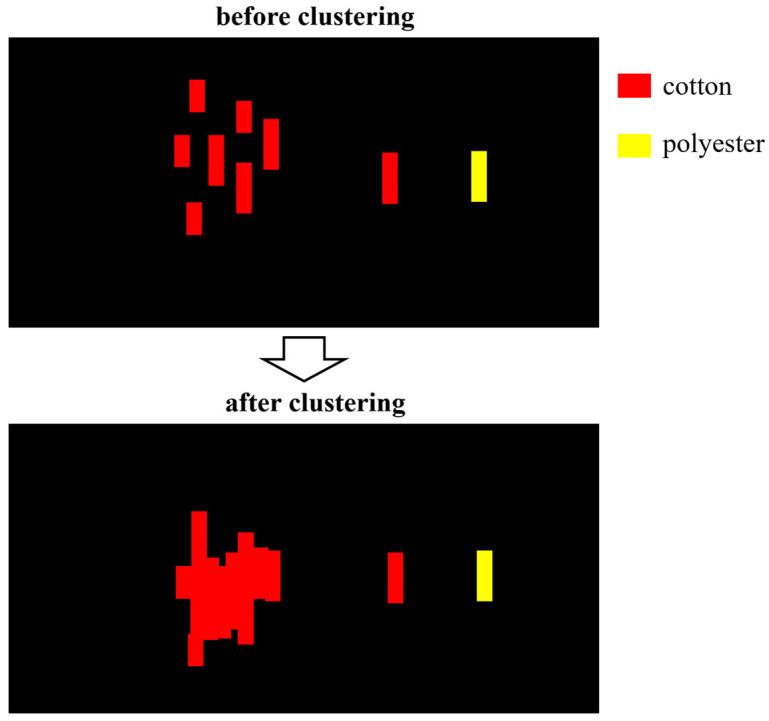
Clustering diagram.

**Figure 10:**
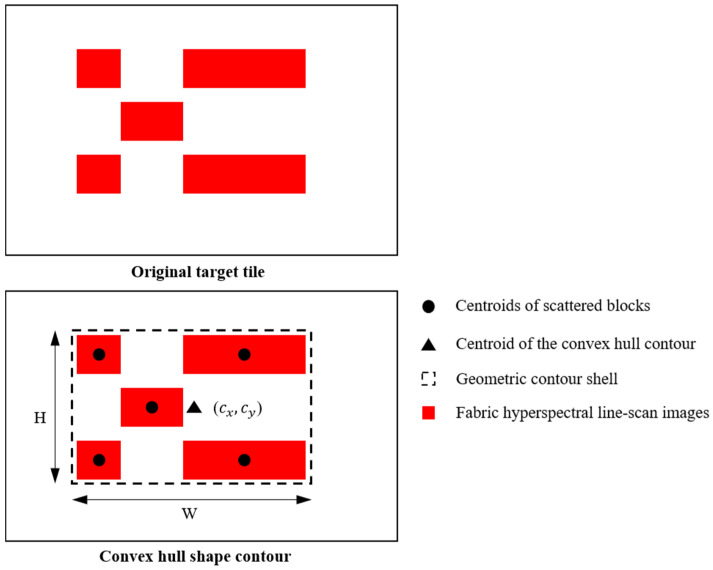
Convex hull calculation diagram.

The system compares it with the center positions of all current active regions. If the center distance is below a set threshold or the horizontal ranges of the row segments overlap, it is assigned to the corresponding region; otherwise, a new region is created to represent a new textile target. Utilizing this lightweight row segment association strategy, the system can stably construct the continuous structure of textiles under row-by-row scanning conditions, as illustrated in Figure 9. When an active region does not receive updates for several consecutive rows, the system determines that the corresponding target has completely passed the scanning range and triggers the region reconstruction process. Suppose the region contains H rows in total; its pixel set can be represented as:

(10) $$P=\left\{(x_{i},y_{i})\left|s_{i}\leq x_{i}\leq e_{i}\right.\right\}$$

where *y_i_* is the scan row number where the pixel resides. The system then calculates the convex hull of set *P* to obtain a compact bounding contour of the target, as shown in Figure 10:

(11) H=ConvexHull(P)

The region centroid is calculated based on image moments:

(12) $$\left(c_{x},c_{y})=(\frac{M_{10}}{M_{00}},\frac{M_{01}}{M_{00}}\right)$$

Considering the fixed proportional relationship between the horizontal pixel position in the hyperspectral image and the physical width of the conveyor belt, the horizontal centroid position can be mapped to the physical coordinate system:

(13) $$y_{mm}=c_{x}\cdot \frac{W_{mm}}{W_{px}}$$

where *W_mm_* represents the effective width of the conveyor belt, and *W_px_* is the horizontal pixel width of the hyperspectral image.

The physical displacement along the conveyor direction is estimated from the timestamp difference between the first and last rows of the region, combined with the conveyor belt speed:

(14) $$x_{mm}=(t_{1}-t_{0})\cdot v$$

where *t_0_* and *t_1_* represent the timestamps of the starting and ending scan rows of the region, respectively.

**Figure 11:**
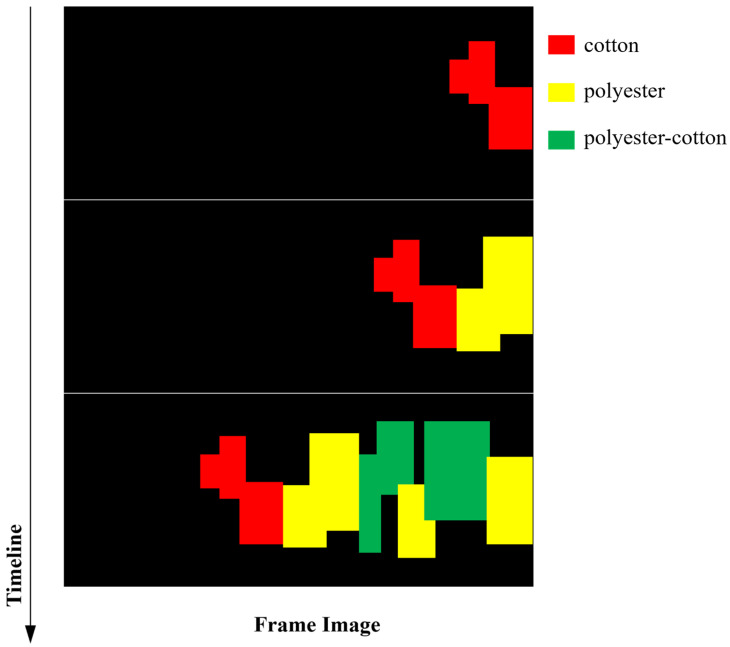
Schematic diagram of hyperspectral stitching.

After completing region stitching and physical position calculation, the hyperspectral host computer generates a structured material information data block based on the reconstruction results (including contour, area, centroid coordinates, material category, etc.). Finally, the system sends this data to the sorting control host computer via TCP/IP Socket in JSON format for subsequent RGB image detection and material-color matching. This stitching result can be used for visualization and provides critical input for subsequent bimodal spatial association and information fusion (Figure 11).

### 2.7. Material and Color Information Association and Matching Mechanism

**Figure 12:**
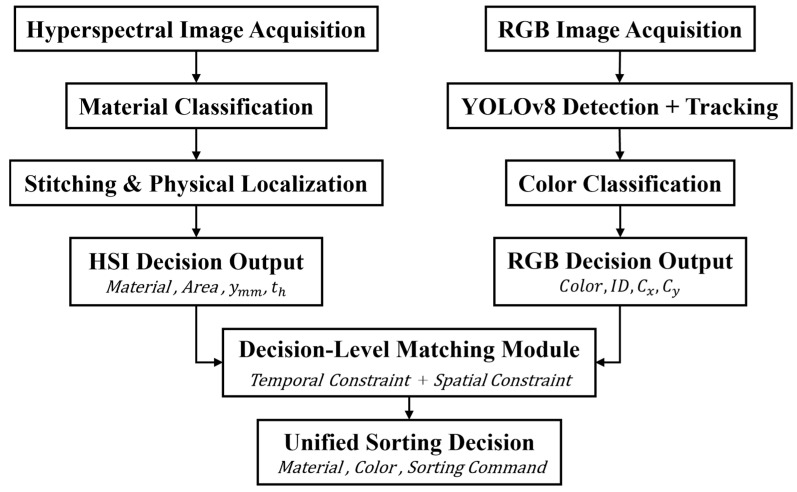
Material–Color Association and Matching Workflow.

The sorting host computer integrates hyperspectral material recognition results and RGB-based color detection results to generate a unique and consistent sorting decision for each fabric piece. From the perspective of multimodal information fusion, a decision-level fusion strategy is adopted, in which the hyperspectral subsystem and the RGB vision subsystem independently complete material classification and color-based target detection, respectively. The correspondence between fabric pieces detected by the two modalities is established through spatiotemporal consistency constraints, rather than through shared raw features or joint feature representations, as shown in Figure 12.

After hyperspectral image stitching is completed, the hyperspectral host computer continuously transmits structured material information data blocks to the sorting host computer. Each data block contains the material category, stitched region area, lateral physical centroid coordinate $\;y_{mm}$, and timestamp $t_{h}$ These data are buffered in a queue denoted as material_buffer. In parallel, the RGB host computer performs real-time target detection on the conveyor belt using YOLOv8n and employs a greedy association strategy based on maximum IoU to generate stable target trajectories. For each tracked target, the RGB system outputs the image center coordinates $\left(c_{x},c_{y}\right)$ along with the corresponding detection timestamp.

#### 2.7.1. Temporal Constraint

To achieve temporal alignment between hyperspectral and RGB detection results under high-speed conveyor belt operation, the theoretical synchronization offset between the two modalities is calculated based on the physical camera spacing and conveyor belt speed. Let *D* denote the distance between the hyperspectral camera and the RGB camera along the conveyor direction, *v* denote the conveyor belt speed, and Δ*t* denote the overall system processing delay. The theoretical temporal offset is given by:

(15) $$t_{sync}=\frac{D}{v}+\Delta t$$

A temporal tolerance term *ϵ* is introduced to account for belt speed fluctuations and processing latency variations. The temporal matching constraint is therefore defined as:

(16) $$\left|t_{now}-t_{h}-t_{sync}\right|\leq \epsilon$$

Only hyperspectral material data satisfying the temporal constraint are considered as candidates for further spatial matching. Here, $t_{\mathrm{now}}$ denotes the current system timestamp at which a target is detected by the RGB vision module, while $t_{h}$ represents the timestamp associated with the hyperspectral material detection result for the same target.

#### 2.7.2. Spatial Constraint

To enforce spatial consistency, the horizontal image coordinate $c_{x}$ obtained from the RGB detection is mapped to the physical coordinate system of the conveyor belt via camera calibration. This mapping converts pixel coordinates into lateral physical coordinates expressed in millimeters:

(17) $$x_{rgb}=\alpha c_{x}+\beta$$

where α is the pixel-to-millimeter scaling factor and β is the lateral offset, both determined through camera calibration.

The spatial matching constraint between the RGB detection result and the hyperspectral material result is then defined as:

(18) $$\left|x_{rgb}-y_{mm}\right|\leq \delta_{s}$$

where $\delta_{s}\;$ denotes the spatial matching tolerance, determined by the combined effects of camera resolution, calibration accuracy, and the typical lateral size of fabric pieces.

It should be noted that although different symbols are used, $x_{\mathrm{rgb}}$ and $y_{mm}$ represent the same lateral physical direction of the conveyor belt, with the notation difference arising from the independent coordinate conventions of the RGB area-scan camera and the line-scan hyperspectral imaging system.

#### 2.7.3. Joint Matching Decision

A valid material–color correspondence is established only when both the temporal constraint in Equation (16) and the spatial constraint in Equation (18) are satisfied. Upon successful matching, the hyperspectral material category $C_{\mathrm{hyper}}\;$ and the RGB color category $C_{\mathrm{rgb}}$ are jointly assigned to the same tracked target entry.

Finally, the system generates a grasping instruction only when the fused material–color attributes satisfy the user-defined sorting strategy (e.g., cotton + white, polyester + blue) and when the target center passes the predefined scan trigger line. Each instruction contains the target’s final spatial coordinates, material category, color category, and unique ID, and is transmitted to the PLC via the OPC UA protocol to enable precise and reliable sorting based on bimodal information fusion.

### 2.8. System Integration

This section outlines the overall workflow of the waste fabric sorting system.

**Figure 13:**
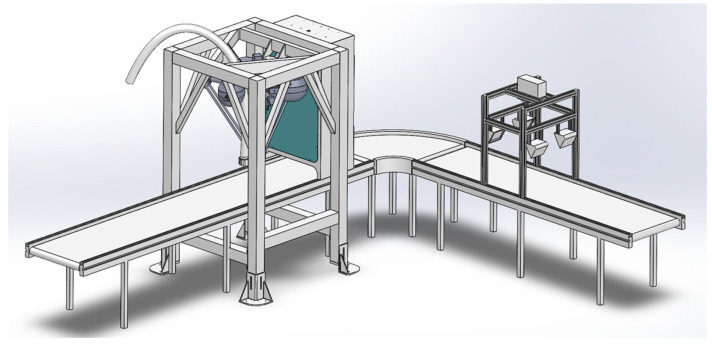
System software structure diagram.

**Figure 14:**
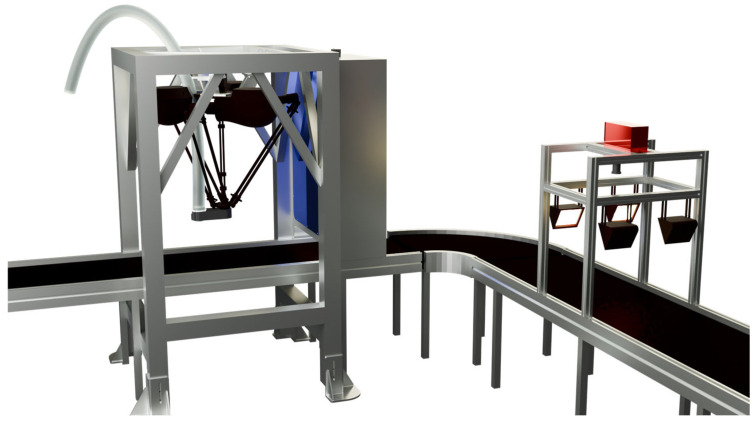
System rendering.

As shown in Figure 13 and Figure 14, the system uses the conveyor belt as the carrier, sequentially performing hyperspectral material detection, RGB color detection, and target localization. The sorting host computer fuses and matches the multi-source detection results, generating sorting decisions. Finally, the sorting instructions drive a parallel Delta manipulator, which utilizes a vacuum suction device to pick and sort the textiles in the grasping area. The overall implementation workflow of the proposed system is summarized in Algorithm 1.
**Algorithm 1**. Overall implementation workflow of the proposed HSI–RGB fabric sorting system.Input:  Hyperspectral scanline data  RGB image frames  Conveyor belt encoder pulsesOutput:  Sorting command with associated material and color labels 1: Acquire hyperspectral scanlines using a line-scan camera. 2: Extract one-dimensional spectral vectors and apply normalization. 3: Classify material using the trained 1D-CNN model. 4: Stitch consecutive scanlines based on timestamp continuity. 5: Detect textile targets in RGB frames. 6: Track targets across frames using IoU-based association. 7: Align hyperspectral and RGB results using temporal and spatial constraints. 8: Trigger sorting based on encoder pulses when the target reaches the predefined position. 9: Transmit sorting commands to the PLC via OPC UA communication.

#### 2.8.1. Multi-Threaded Module Integration

**Figure 15:**
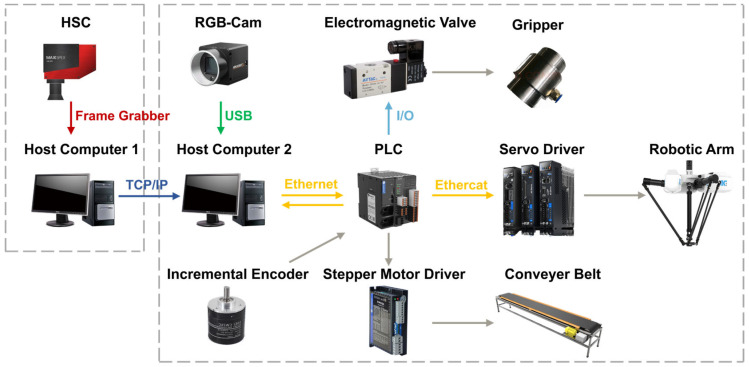
Connection schematic diagram.

**Figure 16:**
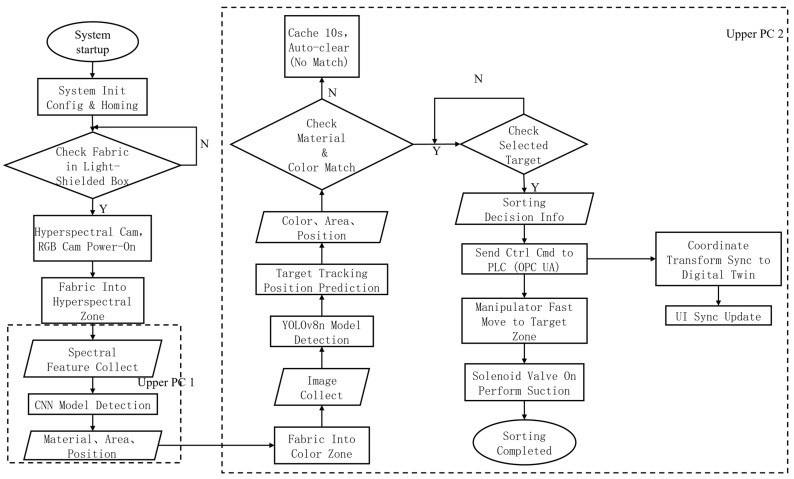
System flowchart.

To meet the requirements of parallel processing of multiple sensors and real-time sorting control in high-speed conveyor belt scenarios, this system adopts a multi-threaded software architecture, decoupling and integrating functional modules such as hyperspectral detection, RGB visual detection, information fusion, and sorting control. The overall system structure and workflow are shown in Figure 15 and Figure 16. Each functional module runs as an independent thread and achieves collaborative work through shared data structures and communication interfaces, ensuring the system’s real-time performance and stability during continuous operation.

During system startup, all core threads are uniformly initialized. Each thread maintains an independent loop during operation, responsible for tasks such as data acquisition, target detection, information processing, and control command issuance. Through multi-threaded parallel execution, the system avoids delays caused by blocking in a single processing pipeline, improves overall throughput efficiency, and enhances adaptability to complex industrial scenarios.

During system operation, hyperspectral detection results and RGB visual detection results converge through a unified data exchange mechanism and are fused for sorting decisions within the host computer. The final sorting instructions are sent to the slave controller via industrial communication protocols, and the actual grasping and sorting operations are performed by the actuators. Logical synchronization between threads is maintained through status flags and queue mechanisms, ensuring the orderliness and reliability of the system operation.

Based on this, the following subsections will introduce the functional division, operational logic, and role within the overall sorting process of each core thread in the system.

#### 2.8.2. Hyperspectral Data Processing Thread

The hyperspectral data processing thread runs on the first host computer. It is primarily responsible for data acquisition from the line-scan hyperspectral camera, spectral feature extraction, and generation of material identification results. This thread continuously receives scan row data output by the camera through a high-speed acquisition interface and preprocesses the raw spectral signals, including noise suppression, normalization, and stable region selection.

After parsing the spectrum of a single row, the thread dynamically combines consecutive scan rows based on a row-by-row association and region stitching algorithm, reconstructing the complete hyperspectral region of the textile along the conveyor belt direction. Simultaneously, the thread invokes the trained hyperspectral material classification model to perform material discrimination on the target region and calculates corresponding spatial information such as contour, area, and centroid.

When a target completes stitching and passes through the scanning area, this thread encapsulates the structured material information data in JSON format and sends it in real-time to the sorting host computer via TCP/IP Socket, providing reliable material and location information input for subsequent color recognition and information fusion.

#### 2.8.3. RGB Image Detection and Target Tracking Thread

The RGB image detection and target tracking thread runs on the second host computer. It is responsible for real-time visual detection, color classification, and multi-frame target tracking of textiles on the conveyor belt. This thread continuously acquires image data from the high-speed RGB camera and performs target detection and color recognition inference on each frame.

The detection stage outputs bounding boxes and color categories for textiles based on a lightweight YOLO network. To ensure consistency of targets across consecutive frames, the thread introduces a greedy association strategy based on maximum IoU to match detection results across multiple frames, assigning a unique ID to each target, thereby forming stable target trajectories.

During the target tracking process, the thread continuously updates the pixel-level center position of targets and maps it to the conveyor belt plane coordinate system using camera calibration parameters. The processes of detection, tracking, and coordinate transformation are all completed within the same thread to reduce inter-thread communication delays and improve overall real-time performance.

#### 2.8.4. Material and Color Information Fusion and Matching Thread

The material and color information fusion and matching thread is responsible for the unified management and spatial matching of detection results from the hyperspectral thread and the RGB visual thread. This thread uses the conveyor belt coordinate system as a common reference framework to align target information obtained from different modalities.

The thread maintains a cache queue for hyperspectral material information, managing received material results with temporal and spatial constraints. It also receives target positions, color categories, and target IDs output by the RGB detection thread. Based on the target’s spatial position along the conveyor belt direction and its running time, the thread completes one-to-one matching of material and color information, generating a complete target attribute description.

This fusion result serves as the sole data source for subsequent sorting decisions, effectively avoiding mismatches caused by asynchronous multi-modal information and providing a stable information foundation for high-speed continuous sorting scenarios.

#### 2.8.5. Sorting Decision and Scheduling Thread

The sorting decision and scheduling thread is responsible for generating specific sorting strategies based on the fused target information. This thread comprehensively considers the target’s material category, color category, and real-time position in the conveyor belt coordinate system to determine whether the target meets the current sorting rules and calculates its timing conditions for entering the grasping area.

During operation, the thread maintains a queue of targets to be sorted and dynamically updates grasping priorities based on conveyor belt speed and target positions. When a target meets the sorting conditions, the thread generates the corresponding sorting instruction and sends the target’s spatial position, category information, and trigger conditions to the communication control module.

Through the scheduling mechanism of this thread, the system can maintain orderly sorting logic when multiple targets enter continuously and reduce the risk of conflicts between actuators.

## 3. Results

### 3.1. Performance Evaluation of the Textile Color Detection Model

**Figure 17:**
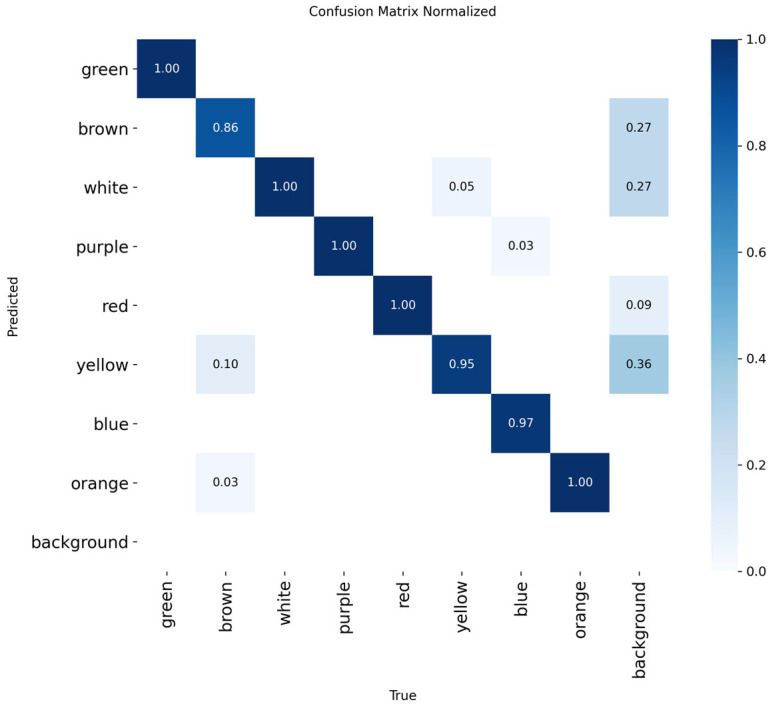
Confusion matrix of the color model.

The proposed textile color detection model was evaluated on the test dataset, and its normalized confusion matrix is shown in Figure 17. The results indicate that the model can effectively distinguish multiple textile color categories, including green, brown, white, purple, red, yellow, blue, and orange. The high proportions along the diagonal of the confusion matrix show that the model achieves high classification accuracy for each color category, demonstrating good overall discriminative performance.

**Figure 18:**
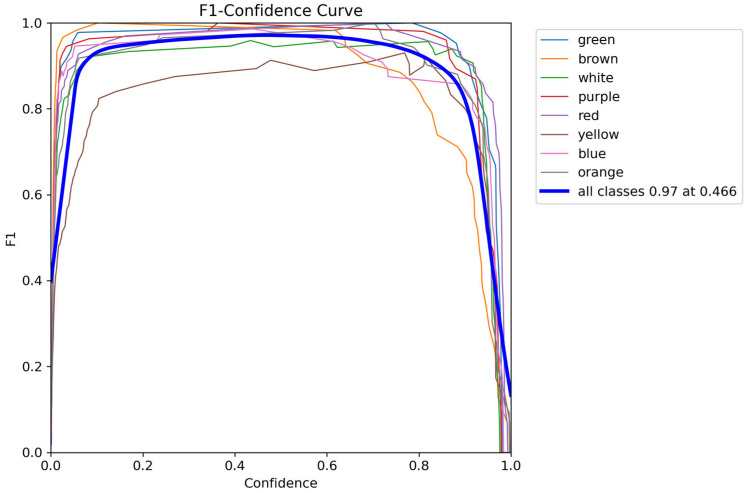
F1-Confidence curve.

To further analyze the model’s comprehensive performance under different confidence thresholds, an F1-Confidence curve was used for evaluation, as shown in Figure 18. The F1 score reflects the balance between precision and recall. Experimental results show that the model maintains high F1 scores across a wide confidence range, indicating good robustness to threshold changes. The model achieves the highest F1 score of approximately 0.97 when the confidence is around 0.47, which can be recommended as the operating threshold for practical system deployment.

Combining the analysis of the confusion matrix and the F1 curve, it can be concluded that the proposed RGB image-based textile color detection model performs well in both classification accuracy and stability, providing a reliable perceptual foundation for subsequent material-color information fusion, matching, and real-time sorting decisions.

#### Robustness to External Environmental Variations

To evaluate the robustness of the color classification module under external environmental variations, additional experiments were conducted based on the RGB-only color detection pipeline, in which color classification is performed solely using the RGB camera without involving hyperspectral information. The trained RGB color classification model was evaluated under both illumination and camera setting variations.

For illumination robustness evaluation, the LED illumination intensity was adjusted using the 8-bit PWM controller described in Section 2.4. Specifically, the PWM duty cycle was varied by ±20% relative to the nominal illumination setting, while all other system parameters, including camera exposure, gain, and model weights, were kept unchanged. In addition, to assess robustness under different camera settings, the exposure time of the RGB camera was varied by ±20% relative to the nominal value while keeping the LED illumination intensity fixed.

Under the nominal condition, the classification accuracy corresponds to the RGB-only results reported in the HSI–RGB ablation study, evaluated using the same trained model and a fixed test subset consisting of 240 fabric samples. For the external robustness evaluation, the trained RGB color classification model was directly applied to the same 240-sample test subset under the modified illumination and exposure settings without retraining. The experimental results show that the color classification accuracy remains stable under moderate illumination and camera parameter variations, indicating that the proposed RGB-based color recognition approach is robust to practical environmental changes commonly encountered in industrial sorting environments (Table 3).

### 3.2. Performance Evaluation of Hyperspectral Material Classification and Image Stitching

**Figure 19:**
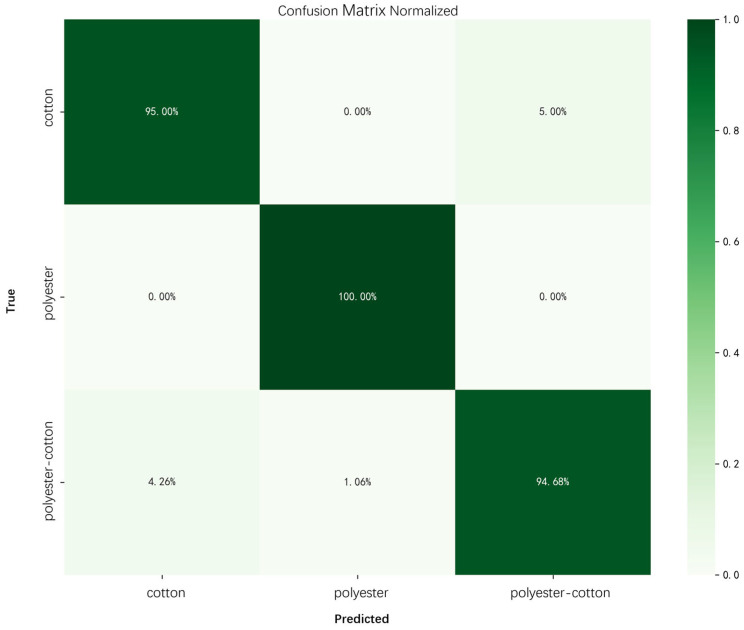
Confusion matrix of the hyperspectral material model.

To verify the effectiveness of the hyperspectral module in the actual sorting system, a comprehensive evaluation of material classification performance and hyperspectral image stitching results was conducted. First, the material classification model was validated on an independent test dataset. The results show that the model can stably identify three common textile material types: cotton, polyester, and cotton-polyester blends, demonstrating good classification and discrimination capabilities (Figure 19).

**Figure 20:**
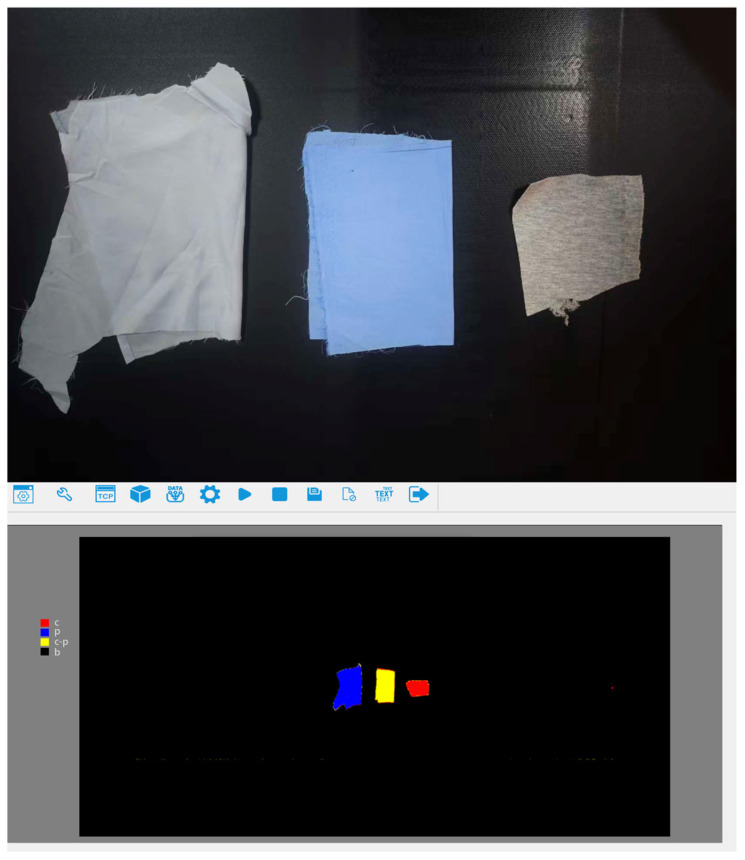
Hyperspectral stitching result.

In addition to material classification performance, the hyperspectral image stitching results were analyzed considering the line-by-line acquisition characteristic of the line-scan hyperspectral camera. Figure 20 shows an example of the stitching result for a typical textile target. It can be observed that, through the row-by-row region association and dynamic buffering mechanism, the system can stably stitch consecutive scan rows into a complete 2D hyperspectral region. The target contour is continuous and clear, without obvious breaks or misalignment.

Further analysis indicates that the centroid positions and size information of the stitched regions show good consistency, providing a stable spatial reference for subsequent RGB visual detection and material-color information matching. The above results validate the stability and engineering usability of the proposed hyperspectral image stitching algorithm in high-speed conveyor belt scenarios.

### 3.3. Ablation Study on the Spatiotemporal Material–Color Matching Mechanism

To identify the primary performance bottleneck of the proposed sorting system, an ablation study was conducted by comparing three configurations: RGB-only, HSI-only, and HSI + RGB with the proposed matching mechanism. All experiments were performed on the same test dataset under identical operating conditions, including conveyor belt speed, illumination, and triggering strategy.

#### 3.3.1. RGB-Only Configuration

In the RGB-only configuration, sorting decisions were generated solely based on the color recognition results provided by the RGB vision module. The hyperspectral camera and material classification module were disabled. Experimental results show that the RGB-only configuration achieves stable and high sorting accuracy, indicating that RGB-based color detection is robust under the tested industrial conditions and does not constitute a major performance bottleneck for the system.

#### 3.3.2. HSI-Only Configuration

In the HSI-only configuration, sorting decisions were based exclusively on hyperspectral material classification results obtained using the 1D-CNN model. It should be noted that, although color recognition was disabled in this configuration, the RGB camera was still used for target detection and localization to ensure consistent triggering and grasping accuracy across all ablation settings. The RGB color classification results, however, were not involved in the sorting decision.

Compared with the RGB-only configuration, the HSI-only configuration exhibits a noticeable reduction in sorting accuracy. The main error sources can be attributed to spectral similarity between blended fabrics, instability of spectral measurements near fabric boundaries in line-scan acquisition, and the sensitivity of hyperspectral signals to motion-related effects under high-speed conveyor operation. These results indicate that hyperspectral material classification represents a critical performance-limiting stage in the current system.

#### 3.3.3. HSI + RGB Configuration with Matching

In the full HSI + RGB configuration, both hyperspectral material recognition and RGB-based color detection were enabled and integrated through the proposed spatiotemporal matching mechanism. Sorting decisions were generated only when material and color information were successfully associated for the same fabric piece.

The results show that the HSI + RGB configuration enables more comprehensive sorting by jointly considering material and color attributes (Table 4). However, the overall sorting accuracy remains constrained by the performance of hyperspectral material classification. Errors originating from the hyperspectral stage propagate to the fused system, even though RGB-based localization and color detection remain stable.

#### 3.3.4. Discussion

Overall, the ablation results demonstrate that the current performance bottleneck of the proposed system lies in hyperspectral material detection rather than in RGB-based localization, color detection, or the material–color matching mechanism. While RGB perception provides reliable target localization and color information, limitations in hyperspectral material classification dominate the system-level performance. These findings suggest that future improvements should primarily focus on enhancing hyperspectral material recognition to further improve overall sorting accuracy.

### 3.4. Integrated System Testing

**Figure 21:**
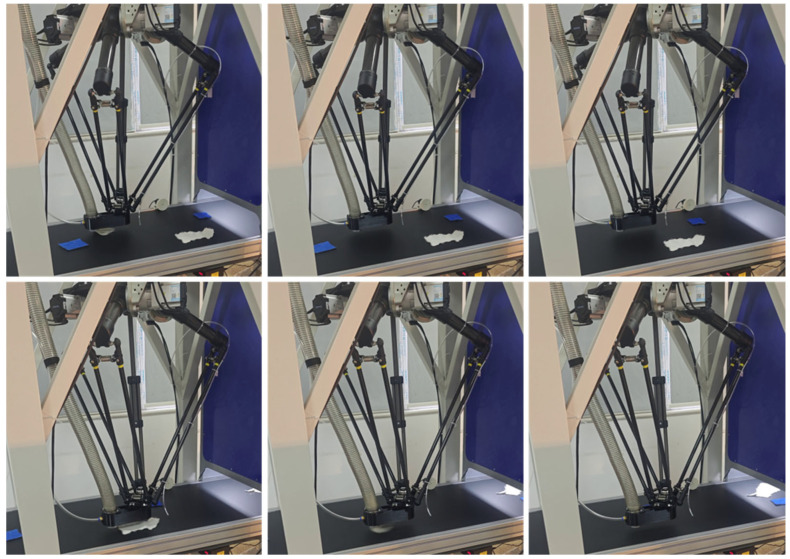
Example of the manipulator grasping process during continuous waste fabric sorting experiments. From (**left**) to (**right**), the images show the textile entering the grasping area, target locking, and the target suction process, reflecting the system’s stable sorting capability under continuous incoming material conditions.

To verify the overall performance of the proposed waste fabric sorting system under actual operating conditions, a complete experimental platform was built, and continuous sorting experiments were conducted. Each experiment consisted of multiple sorting trials performed under identical system configurations, with textile samples randomly placed onto the conveyor belt at irregular time intervals to simulate realistic waste inflow conditions. The system sequentially performed hyperspectral material detection, RGB color recognition, information matching, and sorting decision-making, with a Delta manipulator executing the grasping and sorting operations (Figure 21).

As shown in Table 5, the system maintains a high sorting success rate for pure cotton and pure polyester materials, reaching 95.0% and 97.5%, respectively. These results indicate that the material classification method based on near-infrared hyperspectral features can stably characterize the spectral differences in single-fiber materials, making it suitable for online material identification tasks in high-speed sorting scenarios.

In contrast, the sorting success rate for cotton–polyester blended materials shows a noticeable decline to 85.0%. This is mainly attributed to the fact that blended fabrics contain both cotton and polyester components in their spectral signatures, which vary continuously with the blend ratio. Under the current modeling approach focused on qualitative material classification, samples with extreme composition ratios tend to exhibit spectral characteristics closer to pure cotton or pure polyester, leading to increased confusion during discrimination.

To further analyze this limitation, an additional controlled experiment was conducted by restricting cotton–polyester blended samples to a medium composition range (40–60% cotton content). The results show a clear improvement in sorting accuracy within this range, indicating that the primary source of misclassification originates from blends with extreme component ratios rather than an inherent inability of the system to handle blended materials.

**Figure 22:**
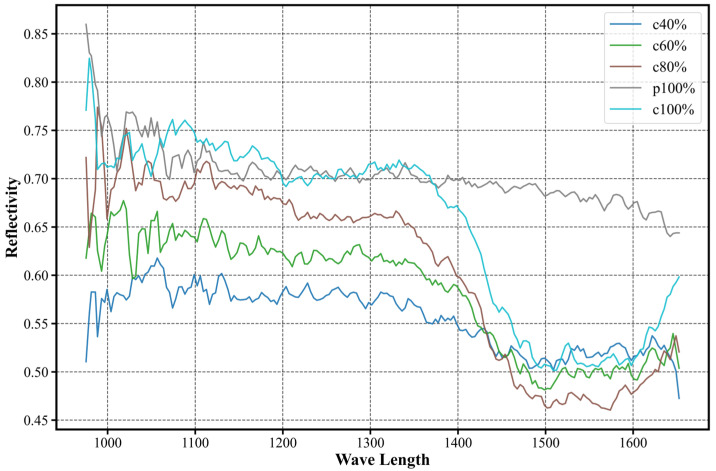
Hyperspectral reflectance comparison of pure and blended textile fabrics.

To further substantiate this interpretation from a spectral perspective, representative hyperspectral reflectance curves of cotton–polyester blended fabrics with different cotton contents (40%, 60%, and 80%), together with pure cotton (100%) and pure polyester (0% cotton), are compared in Figure 22.

As the cotton content decreases, the overall spectral response of the blended fabrics progressively shifts toward that of pure polyester, exhibiting a continuous transition rather than forming a distinct intermediate spectral pattern.

In particular, blends with lower cotton content show spectral curves that closely resemble those of pure polyester across most wavelength bands, providing direct spectral evidence that blended samples with extreme composition ratios are prone to being confused with the dominant parent material under a qualitative classification framework.

From the experimental results, under the same material condition, the sorting performance for samples of different colors shows little variation, indicating that color changes do not significantly impact the system’s sorting results. This aligns with the system’s design philosophy of separately modeling hyperspectral and RGB information for fusion-based decision-making. Material discrimination primarily relies on spectral features, while color information participates in subsequent sorting decisions as an independent dimension. The two can maintain good independence from each other in actual operation.

Synthesizing the above results, it can be concluded that the dominant factor affecting the current system’s sorting performance lies in the spectral complexity of blended materials rather than color variations.

These findings provide a clear experimental motivation for future research focusing on blend ratio estimation, continuous-label modeling, or fine-grained spectral unmixing approaches, which are expected to further enhance the system’s capability in handling complex blended waste fabrics.

### 3.5. Sensitivity Analysis of Triggering and Matching Parameters

To quantitatively evaluate the robustness of the triggering and matching strategy, sensitivity experiments were conducted on the scanline height, spatial distance tolerance $\delta_{s}$, and temporal tolerance ε.

All experiments were performed under controlled conditions using 100 white pure cotton textile samples, which were intentionally selected to minimize the influence of color variability and material ambiguity, allowing the effect of parameter variation to be evaluated in isolation.

#### 3.5.1. Scanline Height Sensitivity

The scanline height sensitivity experiment was conducted under RGB-only detection, without hyperspectral involvement.

A total of 100 white textile pieces were tested, and the evaluation metric was defined as the success rate of the sorting action, i.e., whether each fabric piece triggered exactly one valid sorting command.

Three scanline heights (40, 70, and 100 pixels) were evaluated to analyze the sensitivity of the triggering mechanism. When the scanline height was set to 40 pixels, the narrow triggering region provided high temporal precision but was highly sensitive to bounding-box jitter and frame-level discretization. In practice, a fabric piece could pass through the triggering region between consecutive frames, resulting in no frame fully intersecting the scanline and consequently missed trigger events. Increasing the scanline height to 70 pixels significantly improved robustness by ensuring that at least one frame intersected the triggering region for each fabric piece, yielding the highest sorting success rate. In the early stage of system development, when neither target tracking nor a task queue mechanism was employed, a wider scanline could occasionally lead to repeated triggering; however, this issue has been resolved in the current system implementation. Nevertheless, excessively increasing the scanline height may still introduce temporal uncertainty and reduce the positional precision of the downstream sorting mechanism. When the scanline height was further increased to 100 pixels, although missed triggers were effectively eliminated, the overly thick triggering region introduced temporal ambiguity, occasionally resulting in delayed or overlapping trigger events. Based on these observations, a scanline height of approximately 70 pixels was selected as the optimal trade-off between robustness and temporal accuracy.

#### 3.5.2. Temporal Tolerance Sensitivity

As introduced in Section 2.7.1, the temporal tolerance ε is employed to compensate for temporal misalignment between hyperspectral and RGB detection results caused by belt speed fluctuations and system latency. In this section, ε was varied over a wider range from ±100 ms to ±500 ms to evaluate the robustness of the temporal matching success rate.

When ε was set below 100 ms, the temporal matching success rate decreased noticeably. This degradation can be attributed not only to normal system latency fluctuations but also to practical uncertainties in conveyor operation, including minor belt speed variations, measurement errors in time stamping, and additional timing disturbances introduced by curved conveyor segments along the transport path. Increasing ε to the range of 200–400 ms yielded stable temporal matching performance, indicating strong robustness to such timing uncertainties. However, excessively large ε values increased the likelihood of incorrect temporal associations when multiple fabric pieces were present simultaneously, as the expanded temporal windows began to overlap. Based on the robustness–discrimination trade-off observed in the experiments, ε was set to ±200 ms in the final system configuration.

#### 3.5.3. Spatial Distance Tolerance Sensitivity

The spatial distance tolerance experiment was conducted under combined RGB and hyperspectral detection using 100 white pure cotton textile pieces. In this experiment, only samples that had been successfully matched under the temporal constraint were considered, and a trial was regarded as successful when the corresponding color–material in-formation was correctly associated through spatial matching. The spatial tolerance $\delta_{s}$ was evaluated at 10, 30, 50, and 70 mm to analyze its impact on spatial matching robustness.

When $\delta_{s}$ was set to 10 mm, the spatial matching process became sensitive to camera calibration noise and belt speed estimation errors, resulting in a reduced spatial matching accuracy. Increasing $\delta_{s}$ to 30–50 mm significantly improved robustness, yielding stable spatial matching performance by providing sufficient tolerance for system uncertainties without introducing frequent cross-piece mismatches. However, when $\delta_{s}$ was further increased to 70 mm, the enlarged spatial matching window increased the risk of incorrect associations, particularly when fabric pieces were spatially close. Considering both robustness and discrimination capability, a spatial distance tolerance of $\delta_{s}$ = 30 mm was adopted in the final system configuration. The overall sensitivity analysis results are summarized in Table 6.

### 3.6. End-to-End Runtime Analysis

To evaluate the real-time performance of the proposed textile sorting system, an end-to-end runtime analysis was conducted from a system engineering perspective. In this study, the end-to-end system latency is defined as the computational and communication time required to complete perception, decision-making, and command transmission, excluding the physical transport time of textile targets on the conveyor belt.

Specifically, the system latency is measured from the moment a textile target first enters the hyperspectral sensing region to the issuance of the corresponding sorting command by the PLC. This latency includes hyperspectral data acquisition and material inference, RGB-based color detection and tracking, cross-modal matching between hyperspectral and RGB results, inter-host TCP/IP communication, and OPC UA-based PLC command transmission.

It should be noted that, for an individual textile item, the end-to-end detection and decision process follows a sequential workflow. Therefore, the system latency is evaluated as the linear sum of the runtimes of individual processing and communication modules.

Table 7 summarizes the average runtime of the major system modules under nominal operating conditions, including hyperspectral processing, RGB detection and tracking, RGB–HSI matching, network communication, and PLC response. The results indicate that each module operates within a bounded time interval suitable for online execution.

In addition to end-to-end latency, the throughput of the proposed system is considered from a system-level perspective. Throughput is primarily determined by the physical characteristics of the conveyor belt, including its effective width and transport speed, rather than by the computational capacity of the perception modules. For a conveyor belt with an effective width of approximately 0.7 m operating at a speed of 1.0 m/s, and textile items with an average projected area of about 120 cm^2^, the achievable throughput is on the order of 10^2^ textile items per minute. This estimation accounts for practical feeding conditions, including item spacing and non-ideal packing on the belt, and provides a realistic reference for the operating capacity of the proposed sorting system.

#### End-to-End Latency Under Different Belt Speeds and Loads

To evaluate the robustness of the proposed system under different operating conditions, the end-to-end system latency was analyzed at conveyor belt speeds of 0.5 m/s, 1.0 m/s, and 1.5 m/s, as well as under varying system loads characterized by the number of textile items simultaneously present in the sensing region.

The experimental results indicate that the end-to-end system latency remains nearly constant across the evaluated belt speeds. This behavior is expected, as the computational and communication workloads associated with processing an individual textile item—including hyperspectral material inference, RGB-based detection and tracking, cross-modal matching, networking, and PLC command transmission—are independent of the conveyor belt speed. Changes in belt speed primarily affect the physical transport time and item arrival rate, rather than the processing time required for a single item.

When multiple textile items enter the sensing region simultaneously, a slight increase in end-to-end latency is observed. This increase is mainly attributed to sequential processing and scheduling overhead when handling multiple targets. However, the latency growth remains limited, and no significant degradation or backlog accumulation is observed under the tested load conditions. These results demonstrate that the proposed system maintains stable and predictable latency performance under different belt speeds and moderate target congestion.

### 3.7. Spectral Characteristics of Black Fabrics Under Hyperspectral Imaging and Engineering Implications

**Figure 23:**
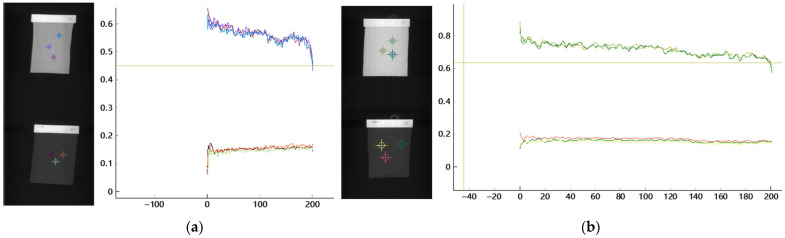
Hyperspectral reflectance curves of fabrics with different colors: (**a**) polyester fabrics; (**b**) cotton–polyester blended fabrics. The horizontal axis represents the sequential index of the 202 hyperspectral bands (0–201) rather than the physical wavelength, and the vertical axis denotes the normalized spectral reflectance. This representation is adopted to preserve the band order and enhance visual clarity, as the center wavelengths of the bands are not evenly spaced within the spectral range of 953.975–1652.48 nm due to the grating dispersion characteristics of the hyperspectral sensor.

To further investigate the sensing limitations associated with black fabrics, their spectral characteristics were analyzed using hyperspectral imaging. Figure 23a,b present comparative spectral reflectance curves of black and non-black polyester fabrics, as well as black and non-black cotton–polyester blended fabrics, respectively. As illustrated in these figures, black fabrics exhibit consistently low reflectance across the entire measured spectral range, with no pronounced absorption or reflection features that can be reliably exploited for material or color discrimination. In contrast, non-black fabrics show higher overall reflectance levels and more distinguishable spectral variations, which provide informative cues for hyperspectral-based analysis.

The observed spectral behavior indicates that black fabrics absorb a large proportion of incident light across both visible and near-infrared wavelengths, resulting in weak hyperspectral responses and reduced signal-to-noise ratios. Consequently, black fabrics are not only difficult to distinguish from the conveyor belt background in RGB imaging but also present limited discriminative information even under hyperspectral sensing. This limitation originates from the intrinsic optical absorption properties of black materials rather than deficiencies in the proposed hyperspectral analysis or classification algorithms.

From an engineering perspective, several practical remedies can be considered to mitigate the limitations associated with black fabric handling in future system extensions. These include modifying the conveyor belt material or surface properties to increase spectral contrast, employing multi-angle or higher-intensity illumination strategies to enhance effective reflectance, and integrating complementary sensing modalities such as active near-infrared illumination or polarization-sensitive imaging. With such system-level enhancements, the detectability and separability of black fabrics can be improved without fundamentally altering the core hyperspectral processing framework proposed in this study.

## 4. Conclusions

Focusing on the application requirements for high-speed sorting of waste textiles, this paper proposes a real-time method for fabric material and color sorting based on the fusion of hyperspectral and RGB visual features. Addressing the difficulty of simultaneously recognizing both material and color using a single sensing modality in complex industrial scenarios, this paper, from the perspective of multi-modal perception and information fusion, constructs a technical pathway for detection and sorting suitable for high-speed conveyor belt conditions.

At the methodological level, targeting the imaging characteristics of line-scan hyperspectral cameras, a hyperspectral image stitching and target reconstruction method based on scan row association is proposed. This achieves continuous representation of fabrics along the conveyor direction and provides a stable spatial localization foundation for subsequent RGB image detection. Based on near-infrared hyperspectral data, a one-dimensional convolutional neural network was designed and trained, enabling effective discrimination of typical fabric materials such as cotton, polyester, and cotton-polyester blends. Simultaneously, a lightweight color detection model was constructed based on RGB images, achieving real-time detection and localization of multi-colored fabrics in high-speed motion.

Regarding multi-modal fusion, this paper proposes a material and color information matching mechanism based on temporal and spatial coordinate constraints. It associates and fuses detection results from hyperspectral and RGB vision, thereby achieving unified determination of fabric material and color attributes. Experimental results show that this fusion method achieves high recognition accuracy in sorting tasks for pure cotton and pure polyester fabrics and maintains stable real-time performance under high-speed conveyor belt conditions.

Future work will further focus on quantitative component analysis of blended fabrics, fine-grained modeling of hyperspectral feature representation and enhancing the robustness of multi-modal fusion strategies. This aims to further improve recognition accuracy and applicability in complex textile sorting scenarios.

Overall, the proposed spectral-RGB feature fusion method provides a feasible and effective technical solution for the high-speed, automated sorting of waste textiles.

## Figures and Tables

**Table 1:** Architecture of the proposed 1D-CNN for hyperspectral material classification.

Stage	Layer	Configuration	Output Description
Input	Spectral vector	1 × N	Normalized 1D spectrum
Conv Block 1	Conv1D + BN + ReLU	64 filters, k = 5	Local spectral features
	MaxPool1D	stride = 2	Down-sampling
	Dropout	*p* = 0.3	Regularization
Conv Block 2	Conv1D + BN + ReLU	128 filters, k = 5	Mid-level spectral patterns
	MaxPool1D	stride = 2	Down-sampling
	Dropout	*p* = 0.3	Regularization
Conv Block 3	Conv1D + BN + ReLU	256 filters, k = 3	High-level spectral features
	MaxPool1D	stride = 2	Down-sampling
	Dropout	*p* = 0.4	Regularization
Conv Block 4	Conv1D + BN + ReLU	512 filters, k = 3	Abstract spectral representation
	Adaptive Avg Pool	output = 1	Length-invariant features
	Dropout	*p* = 0.5	Regularization
FC Block	FC + BN + ReLU	512 → 256	Feature compression
	Dropout	*p* = 0.5	Regularization
	FC + BN + ReLU	256 → 128	High-level embedding
	Dropout	*p* = 0.4	Regularization
Output	FC + Softmax	128 → 3	Material probabilities

**Table 2:** Statistics of the textile color classification dataset.

Color Class	Total Samples	Training	Validation	Test
White	28	20	4	4
Red	27	19	4	4
Green	29	21	4	4
Blue	26	18	4	4
Yellow	28	20	4	4
Brown	29	21	4	4
Orange	27	19	4	4
Purple	28	20	4	4
Total	222	158	32	32

**Table 3:** Color classification accuracy under different external environmental conditions.

Illumination Setting	Setting	Accuracy (%)
Nominal (RGB-only baseline)	Standard illumination & exposure	97.92
Illumination variation	−20% LED PWM	96.67
Illumination variation	+20% LED PWM	97.08
Camera setting variation	−20% exposure time	96.67
Camera setting variation	+20% exposure time	98.33

**Table 4:** Ablation study on different sensing configurations.

Configuration	Used Modality	RGB Used for Localization	Material–Color Matching	Sorting Accuracy (%)	Main Error Source
RGB-only	RGB	✓	✗	97.92	Minor color ambiguity under complex illumination
HSI-only	Hyperspectral	✓	✗	91.25	Spectral overlap in blended fabrics
HSI + RGB	Hyperspectral + RGB	✓	✓	88.75	Error accumulation dominated by hyperspectral material classification

Note: All ablation experiments were conducted on the same test set consisting of 240 fabric pieces, including cotton, polyester, and blended fabrics. In the HSI-only configuration, the RGB camera is used solely for target detection and localization, while RGB color classification results are not involved in the sorting decision. Sorting accuracy is defined as the percentage of fabric pieces correctly sorted according to the active decision criteria of each configuration.

**Table 5:** Statistical results of sorting experiments for waste fabrics of different materials and colors.

Material Type	Composition Range	Number of Tests	Number of Successful Sorts	Sorting Success Rate (%)	Main Misclassification Cases
Cotton	100% cotton	80	76	95.0	Misclassified as blend
Polyester	100% polyester	80	78	97.5	Misclassified as blend
Cotton-Polyester Blend	0–100%	80	68	85.0	Confusion with pure classes
Cotton-Polyester Blend *	40–60% cotton	80	74	92.5	Occasional material confusion

* This subset was not treated as an additional material category, but was used solely for controlled performance analysis.

**Table 6:** Sensitivity analysis results for scanline height, temporal tolerance, and spatial distance tolerance.

Parameter Category	Parameter Value	Experimental Condition	Evaluation Metric	Success Rate (%)
Scanline height (pixels)	40	RGB-only detection	Successful triggering and sorting	74
	70	RGB-only detection	Successful triggering and sorting	99
	100	RGB-only detection	Successful triggering and sorting	87
Temporal tolerance ε (ms)	±100	RGB + HSI detection	Temporal matching success rate	67
	±200	RGB + HSI detection	Temporal matching success rate	98
	±300	RGB + HSI detection	Temporal matching success rate	95
	±400	RGB + HSI detection	Temporal matching success rate	88
Spatial tolerance $\delta_{s}$ (mm)	10	RGB + HSI detection	spatial matching success rate	84
	30	RGB + HSI detection	spatial matching success rate	97
	50	RGB + HSI detection	spatial matching success rate	96
	70	RGB + HSI detection	spatial matching success rate	92

**Table 7:** Average runtime of individual system modules under nominal operating conditions.

Module	Description	Average Runtime (ms)	Remarks
Hyperspectral data acquisition & material inference	Line-scan HSI acquisition, preprocessing, and CNN-based material classification	10.4	Includes spectral normalization and inference; executed on HSI host
RGB detection and tracking	RGB-based fabric detection and tracking using YOLOv8n	8.5	Real-time execution on RGB host
RGB–HSI result matching	Temporal–spatial association between HSI and RGB results	3.1	Lightweight matching logic
Inter-host communication	TCP/IP socket transmission of HSI results	2.6	Average one-way transmission delay
PLC command transmission	OPC UA communication and PLC response	8.7	Includes command packaging and acknowledgment
End-to-end system latency	From HSI target entry to PLC command issuance	33.3	Linear timing budget

Note: All reported runtimes represent average values measured over multiple runs under nominal operating conditions.

## Data Availability

The data presented in this study are available on request from the corresponding author.
